# Supplementary material for: Enforced Mutualism Leads to Improved Cooperative Behavior between Saccharomyces cerevisiae and Lactobacillus plantarum
Source: Microorganisms. 2020 Jul 24;8(8):1109. doi: 10.3390/microorganisms8081109 (PMC7464542; doi:10.3390/microorganisms8081109)
Supplement: Supplementary file 1 [file microorganisms-08-01109-s001.pdf]

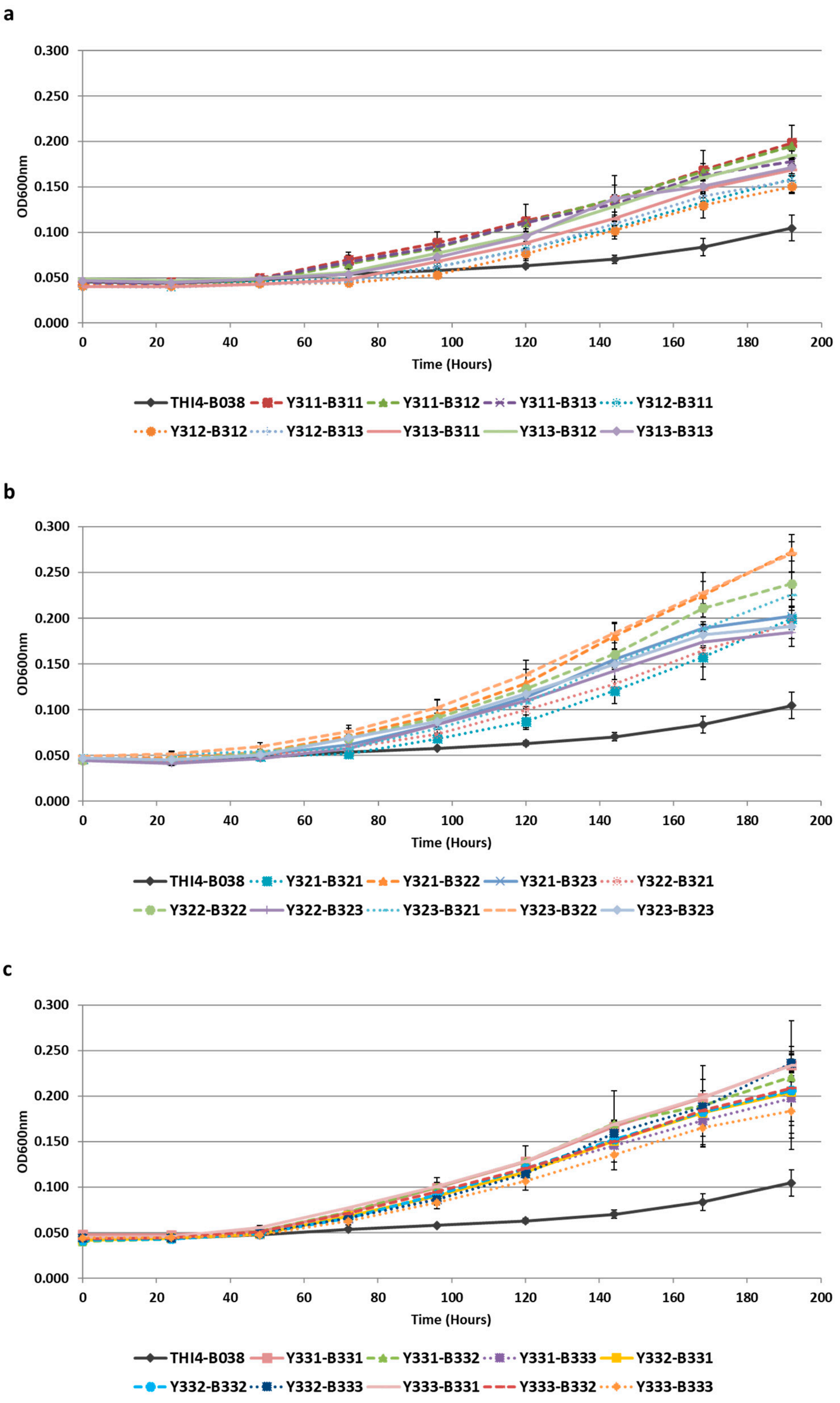

Figure S1. Growth of *S. cerevisiae* BY4742Δ*thi4* and *Lb. plantarum* IWBT B038 evolved pairings in the absence of lysine and isoleucine. THI4-B038 is the growth of the unevolved parental pairing compared to the growth of the evolved pairings from each biological repeat (**a**, **b**, **c**). Data shown are means of triplicates with error bars representing standard deviation.

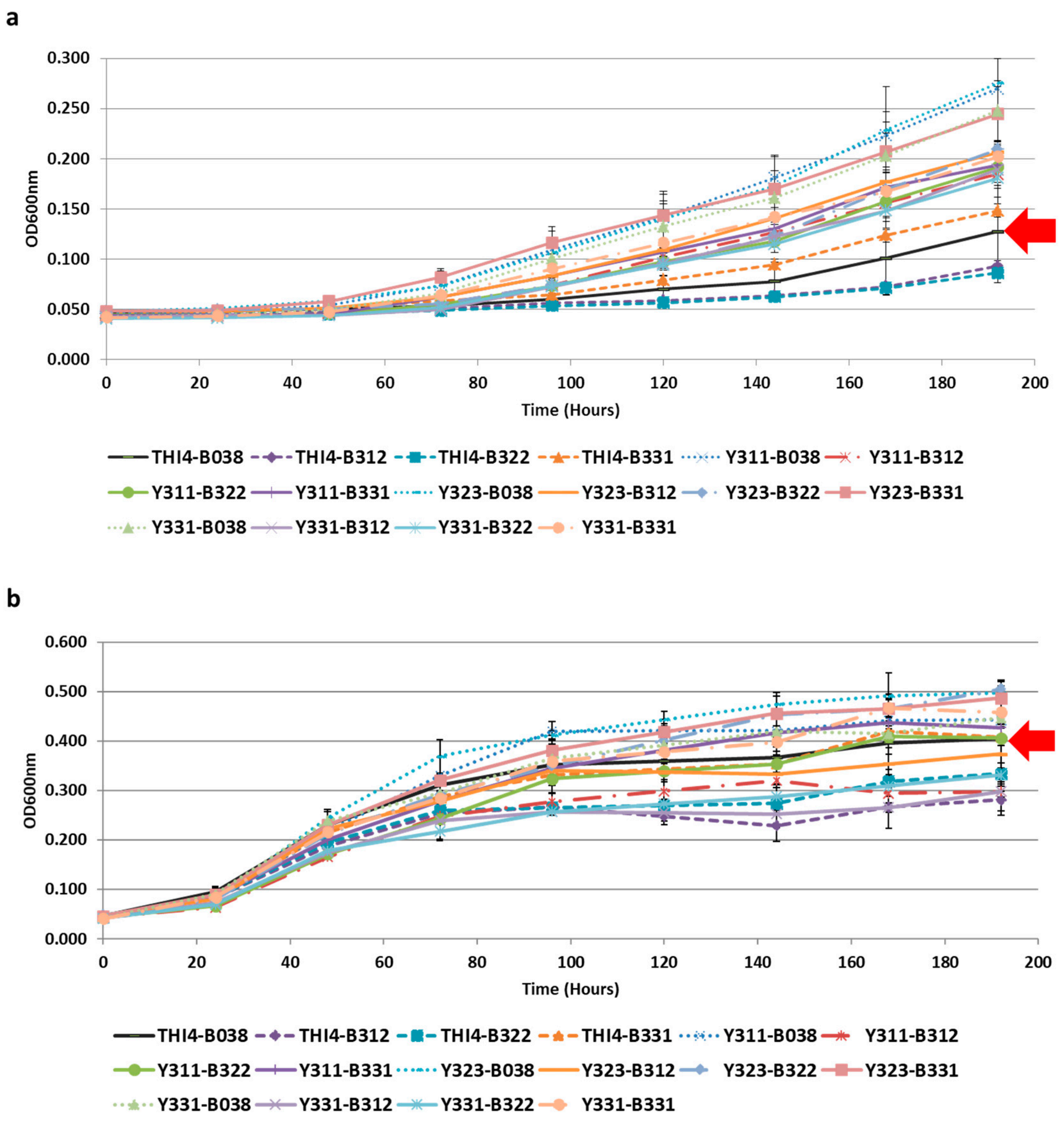

Figure S2. Paired growth of all *S. cerevisiae* BY4742Δ*thi4* and *Lb. plantarum* IWBT B038 evolved isolates in the absence of lysine and isoleucine (**a**) and presence of all amino acids (**b**). Red arrows show the growth of the unevolved parental pairing THI4-B038 (**ab**). Data shown are means of triplicates with error bars representing standard deviation.
